# Loop parallelization in source code for internet of things computing using hybrid heuristic algorithm

**DOI:** 10.1371/journal.pone.0341059

**Published:** 2026-03-27

**Authors:** Bahman Arasteh, Seyed Salar Sefati, Huseyin Kusetogullari, Farzad Kiani, Shahryar Sorooshian, Erfan Babaee Tirkolaee

**Affiliations:** 1 Department of Software Engineering, Faculty of Engineering and Natural Science, Istinye University, Istanbul, Turkiye; 2 Department of Computer Science, Khazar University, Baku, Azerbaijan; 3 Telecommunications Department, Faculty of Electronics, Telecommunications and Information Technology, National University of Science and Technology POLITEHNICA Bucharest, Bucharest, Romania; 4 Artificial Intelligence Research Center (AIRC), College of Engineering and Information Technology, Ajman University, Ajman, UAE; 5 Department of Computer Science, Blekinge Institute of Technology, Karlskrona, Sweden; 6 Data Science Application and Research Center (VEBIM), Fatih Sultan Mehmet Vakif University, Istanbul, Turkiye; 7 Department of Business Administration, University of Gothenburg, Gothenburg, Sweden; 8 Faculty of Engineering and Sustainable Development, University of Gävle, Gävle, Sweden; 9 Department of Industrial Engineering, Istinye University, Istanbul, Turkey; 10 Department of Industrial Engineering and Management, Yuan Ze University, Taoyuan, Taiwan; Xidian University, CHINA

## Abstract

Efficient task scheduling remains a key challenge in High-Performance Computing and Internet of Things (IoT) systems, where the sequential execution of nested loops often limits parallelism. This paper proposes a hybrid approach that dynamically parallelizes nested loops in heterogeneous IoT environments. The suggested method (PSOALS) combines Particle Swarm Optimization (PSO), Genetic Algorithm (GA), and wave-angle scheduling to model nested loops as two-dimensional iteration spaces and minimize communication overhead. By encoding loop iterations as particles and using a dependency-aware fitness function, PSOALS enhances makespan, resource utilization, and scalability. The key contributions of this work include: a dynamic scheduling framework for efficient loop parallelization and dependency management, a wave-angle scheduling mechanism to improve task execution order by balancing load and communication delays, and the integration of mutation and diversity techniques to enhance the quality of the solution. Experimental results across various IoT configurations show that PSOALS outperforms block-based, cyclic, and GA-based scheduling methods in convergence speed, stability, and execution time. The proposed approach offers a scalable and adaptive solution to future IoT challenges, including real-time processing, energy efficiency, and large-scale deployment.

## 1. Introduction

In IoT environments, applications often involve data-intensive operations such as real-time monitoring, sensor data aggregation, and edge-level analytics, which are frequently implemented using nested loop structures. When executed sequentially, these loops can become performance bottlenecks in heterogeneous IoT systems, where processing resources are distributed across cloud, fog, and edge layers [1]. Loop parallelization is therefore essential for exploiting available computational capacity and minimizing execution delays. By transforming nested loops into parallelizable components, task execution can be distributed across multiple cores or nodes. Moreover, effective loop parallelization helps mitigate energy consumption and resource underutilization. A significant challenge in such systems arises from repetition loops, which often dominate execution time. Task scheduling is essential for ensuring efficient execution. Tasks must be appropriately assigned to processors to optimize execution time. This issue is exacerbated in nested loops, where multiple levels of iteration significantly inflate execution times.

Repetition loops, such as those implemented with” for” statements, are critical in IoT systems for executing repeated actions, such as data collection, processing, and transmission across distributed sensors [2]. In IoT environments, programs with nested loops often exhibit increased execution time due to the sequential processing of tasks, particularly when handling large-scale sensor networks or real-time data streams. To overcome these limitations, various scheduling algorithms have been proposed to optimize the execution time of nested loops, enabling faster data handling and improved system responsiveness in IoT architectures.

Time efficiency is paramount in IoT applications, particularly when processing data-intensive tasks with nested loops. These loops frequently account for a significant portion of the system’s execution time, and dependencies between iterations further delay operations [3]. However, iterations without dependencies can be executed in parallel, significantly enhancing performance, which is a critical requirement for IoT systems. This parallel execution is often achieved using multiple processors or edge computing nodes in IoT networks. Additionally, legacy sequential programs designed for earlier IoT implementations can be transformed into parallel versions using advanced interpreters that detect latent parallelism [4]. The scalability and adaptability of previous methods, such as evolutionary algorithms and static scheduling techniques, are limited, particularly in high-dimensional, heterogeneous settings. These approaches frequently require extensive preprocessing or rely on fixed parameters that cannot adjust the workload dynamically. Lower convergence and flexibility are the other drawbacks of the existing methods.

Existing approaches, including Genetic Algorithms (GAs) and static scheduling methods, face limitations in scalability and adaptability, particularly in high-dimensional and heterogeneous environments [5]. These methods often require substantial preprocessing or rely on fixed parameters that fail to adapt to dynamic workload variations. Limited data locality, higher computation cost, complexity in adapting to heterogeneous hardware platforms, challenges in workload adaptation, and high configuration complexity are the main drawbacks of the existing methods. In contrast, the proposed method leverages the collective intelligence of Particle Swarm Optimization (PSO) and GA to refine scheduling solutions iteratively, achieving superior convergence and adaptability. The innovative integration of wave angles as control parameters distinguishes this work by addressing dependencies and balancing workloads across processors effectively.

This work develops a variant of PSO with the Adaptive Loop Scheduling (PSOALS) method to address the challenges of task scheduling in such environments. PSOALS combines PSO with GA and wave-angle scheduling to effectively parallelize nested loops modeled as two-dimensional iteration spaces. This approach ensures the resolution of task dependencies while enabling optimized parallel execution. In the nested loop, instead of iterating row by row, the iteration space is divided into tiles (task blocks). Each task block, or tile, is represented as a particle whose position is iteratively updated according to a fitness function that minimizes makespan and communication overhead. The position of each tile (particle) indicates which processor executes it. Each tile is scheduled independently to improve data locality and parallel execution. A dependence vector represents the relationship between two dependent iterations in a nested loop. Diagonal (wave) execution of the dependent loops, rather than row-by-row execution, enables parallelization. Additionally, wave angle scheduling prioritizes tasks subject to dependency constraints to enhance parallelism and reduce idle processor time. The framework incorporates adaptive mechanisms, such as mutation and diversity enhancements, to mitigate premature convergence and explore higher-quality solutions. The proposed PSOALS method is based on the following assumptions:

1. Loops are fully nested with two levels,
2. Processors are tightly connected,
3. The input consists of tiled iteration spaces,
4. Dependence vectors do not exceed the dimensions of the tiles,
5. A dependency matrix is an n × m matrix, where n indicates the number of loop levels and m stands for the number of dependencies found in the loop,
6. Each element of the matrix shows the dependence direction between loop iterations.

This work makes several significant contributions:

- **Dynamic Scheduling Framework:** Introduction of the PSOALS framework, which models nested loops as two-dimensional iteration spaces, enabling efficient parallelization and dependency resolution in heterogeneous IoT environments.
- **Wave-Angle Scheduling:** Development of a wave-angle scheduling mechanism to optimize task execution order by balancing computational loads, minimizing communication delays, and maximizing parallelism.
- **Fitness-Driven Optimization:** Design of a fitness function that dynamically updates task scheduling, reducing makespan while accounting for dependency constraints and communication overhead.
- **Adaptive Robustness Mechanisms:** Incorporation of mutation and diversity enhancement techniques within the PSOALS framework to prevent premature convergence and facilitate the discovery of high-quality scheduling solutions.

The remainder of this manuscript is organized as follows: Section 2 explains the fundamental concepts; Section 3 presents a review of related work. Section 4 details the developed method. Section 5 evaluates the framework through experiments. Section 6 presents and concludes the research findings, along with a useful outlook for future studies.

## 2. Fundamental concepts

**Fig 1:**
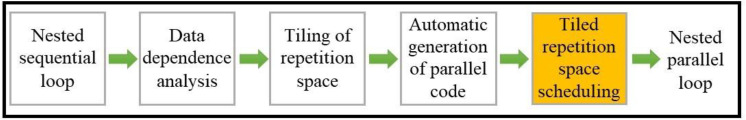
Steps of parallelization of nested loops.

The main steps of nested-loop parallelization are shown in Fig 1. The transformation process, as illustrated in the figure, is vital for optimizing resource utilization and ensuring scalability in modern IoT systems.

Data dependency analysis is the first step in loop parallelization following nested loop detection. In IoT applications, understanding data dependencies is essential for efficiently scheduling tasks across distributed devices [5]. This analysis ensures that tasks are executed in the correct order and optimizes resource usage, reducing delays and improving overall system performance. When two instructions or tasks have no dependency, they can be executed in parallel, enabling faster data processing and reduced latency [6]. Data dependencies, often arising from array usage within loops, must be analyzed and resolved to ensure proper scheduling, particularly in IoT systems handling real-time sensor data or computationally intensive tasks.

Tiling the repetition space for IoT applications is the second step in the loop parallelization process. To enhance parallelization in IoT systems, the repetition space of nested loops is divided into smaller tiles, a process known as tiling. This division reduces communication overhead between IoT nodes or processors and optimizes the distribution of dependent iterations across the network. Efficient tiling is particularly beneficial in IoT scenarios where resources are distributed, and minimizing communication delays is critical for maintaining system performance.

Automatic parallel code generation in IoT Architectures is the next step of loop parallelization. For IoT systems, parallel code is automatically generated for the tiled repetition space, accounting for tile size and shape [7]. Techniques like the wavefront method are particularly effective in these scenarios, ensuring efficient execution of tasks on edge nodes, gateways, or cloud servers. This approach supports scalability and adaptability in IoT environments with dynamic workloads.

**Fig 2:**
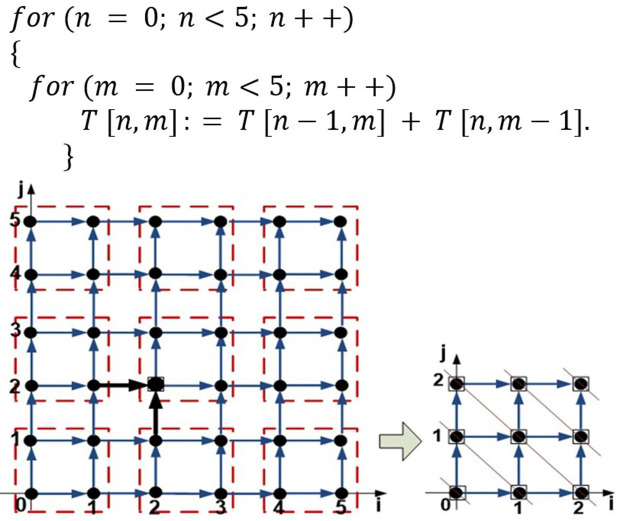
Repetition space and tiled repetition space for data dependency code.


The tiled repetition spaces are scheduled using the wave-front approach to minimize the completion time of all tiles. This scheduling strategy assigns tiles to IoT devices or processors, taking into account data dependencies and communication costs. Wavefront scheduling optimizes task distribution to ensure efficient resource utilization and to meet the stringent performance requirements of IoT systems. The code snippet, represented in Fig 2, illustrates a nested loop with a data dependency, as each element of the 2D array T [n, m] is computed based on values from its previous row (T [n−1, m]) and column (T [n, m−1]). Such dependencies are common in IoT applications that require iterative data processing, such as environmental monitoring and predictive analytics.

$$\begin{array}{c} {}*20cfor\;(n=0;n<5;\;n++)\\ \{\;\;\;\;\;\;\;\;\;\\ \;\;for\;(m=0;m<5;\;m++)\\ \begin{array}{c} \;\;\;\;\;\;\;\;\;T[n,m]:\;=T[n-1,m]+T[n,m-1].\\ \;\;\;\;\;\;\;\;\} \end{array} \end{array}$$

Since scheduling this type of problem is NP-complete, various approximate methods have been proposed. For instance, Fig 2 illustrates the repetition space of a two-level nested loop and its tiled repetition space after dependency analysis. Each point (*n, m*) in Fig 2 represents a loop iteration, while edges indicate dependencies. For example, the iteration (2*,*2) depends on data from (1*,*2) and (2*,*1). The goal is to assign processors to tiles in a way that minimizes execution time while considering communication costs between processors. In the context of nested-loop parallelization, a tile is a subset of loop iterations that must be executed on a processor. The iteration space of nested loops is partitioned into tiles to enhance parallelism, improve cache locality in single processors, and extract coarse-grained parallelism in multiprocessor systems. Tiling aims to minimize inter-processor communication while achieving an optimal allocation of dependent-loop iterations for execution across processors that exchange messages.

**Fig 3:**
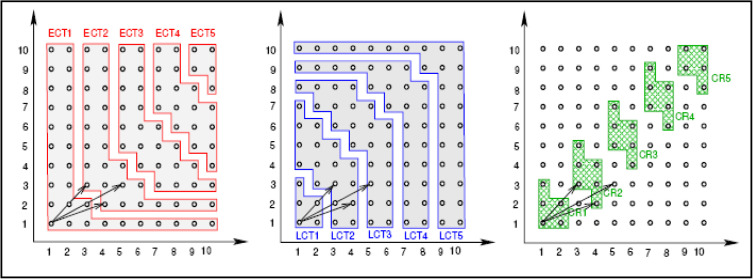
An example of how to calculate the critical path in the loop repetition space [8].

In critical path approaches, the tasks derived from the loop iteration space are organized to ensure balanced workloads across processors while accounting for data dependencies. The critical path consists of loop repetitions that must adhere to specific execution timings. Using this approach, the earliest start time and latest end time of loop iterations are calculated, enabling the identification of iterations on the critical path. Layers within the critical path are defined, with each layer representing a set of iterations that can execute in parallel. Non-critical iterations are distributed among processors to achieve load balance. This strategy is particularly beneficial in IoT systems, where efficient task scheduling is essential to meet real-time constraints, reduce energy consumption, and optimize the use of distributed computational resources. Fig 3 illustrates the layers of the critical path and their potential for parallel execution. In Fig 3, ECT, LCT, and CR are related to the earliest Computation Time, Latest Computation Time, and Critical Region, respectively.

The iteration space is divided into blocks. By grouping iterations into tiles, each tile performs a group of iterations at once, and this improves cache reuse and reduces communication overhead. Tile shape matters because it affects load balancing, parallelism, and data movement efficiency. If tiles are too small, overhead increases and performance decreases. Wavefront parallelization works better with specific tile orientations. In wavefront tiling, tiles are executed in a diagonal wavefront pattern to preserve data dependencies. This technique is commonly used in strong loop-carried dependencies. The iteration space is divided into tiles, typically rectangular or parallelogram-shaped. Tiles are processed in diagonal waves, where each tile depends on previous tiles from earlier time steps. Execution proceeds in parallel along wavefronts, ensuring that dependencies are met while maximizing parallelization. Tile shape directly affects the number of tiles that can be processed concurrently. Regarding the results, wavefront tiling works best when tile shapes allow for near-equal execution time per tile. All in all, tile shape significantly influences optimization (cache efficiency, parallelism, communication overhead), and it should be a decision variable in optimization algorithms.

## 3. Literature review

This section outlines fundamental concepts and related work on optimizing scheduling time for nested loops, a key factor in improving computational efficiency in IoT environments. Athanasaki et al. [9] proposed four scheduling methods: mirror allocation, cyclic allocation, cluster allocation, and retiling scheduling, to organize the tiled repetition space for a cluster system with fixed Symmetric Multi-Processor (SMP) nodes. They implemented these methods using overlapping and non-overlapping plans and evaluated the strengths and weaknesses of each. Re-tiling scheduling performs better in most cases, particularly when the tile shape and count match the number of SMPs. Cluster allocation scheduling minimized execution time by overlapping intra-processor calculations with data transfer time via Direct Memory Access (DMA). This approach grouped tiles for execution on SMP nodes using a wave-generation technique to schedule parallel loops efficiently.

**Fig 4:**
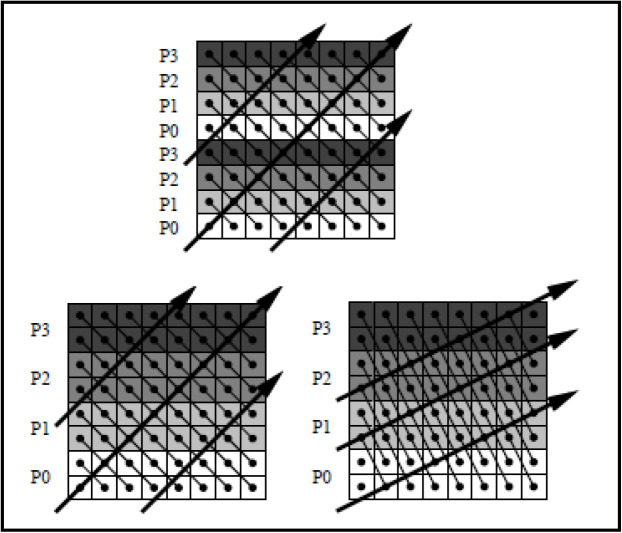
Wavefront parallelization of nested loops using diagonal execution strategy [9].

Saeed Parsa et al. [10] suggested a constraint GA to address the tiling problem in Cartesian spaces for parallel loop execution, focusing on optimizing tile shape and size to minimize inter-tile dependencies and computational overhead. This method represents tiles as parallelepipeds and evaluates fitness based on multiple constraints, including communication, computation, and I/O costs. It initializes populations with random chromosomes, applies selection and crossover operations, and iteratively refines solutions. Advantages include flexibility to handle tiles in higher dimensions and reduced cache misses due to improved tile configurations. However, its computational complexity ($O(n^{\text{3}})$) and reliance on genetic operators may lead to variability in the identification of optimal solutions, particularly for higher-dimensional tiles. Fig 4 depicts this concept.

**Fig 5:**
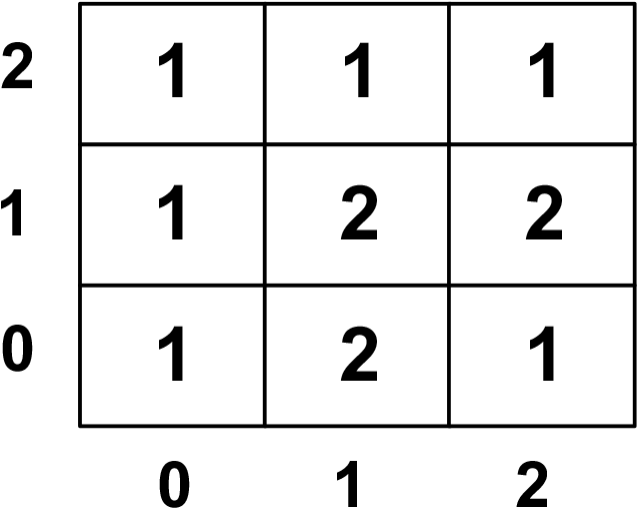
Structure of the two-part chromosome.

AliReza Hajieskandar et al. [11] proposed a GA based on bipartite chromosomes to optimize parallel loop scheduling by reducing the overall execution time of nested loops. The method encodes solutions in two parts: an assignment matrix that maps tiles to processors and a coefficient vector that represents wavefront angles. It applies genetic operators, including crossover and mutation, to iteratively refine the scheduling. Practical evaluations demonstrated that this approach achieves better convergence, greater stability, and higher solution quality than traditional algorithms such as block-based or cyclic scheduling. However, the computational cost of genetic operations and the reliance on heuristic optimizations may limit scalability to very high-dimensional problems. Fig 5 illustrates the concept of GA in chromosome cycling.

Galante et al. [12] proposed an approach to integrate adaptability into parallel programming patterns, enabling applications to dynamically adjust resources in response to changes in computational demand. Their method extended existing parallel programming patterns (such as Single Program Multiple Data (SPMD), Bulk Synchronous Parallel (BSP), MasterWorker, and MapReduce) to include features like dynamic resource allocation, process migration, and elastic scaling. This framework addressed challenges in cloud computing and distributed environments by providing technical guidance and practical tools for managing resource heterogeneity and variability. Advantages include enhanced performance, cost efficiency, fault tolerance, and resource utilization. However, the approach introduced complexity in adapting applications to diverse hardware environments and may require extensive customization for integration with existing systems.

Hao Xu et al. [5] analyzed application co-running interference in throughput-oriented computing systems. Their study utilized modern supercomputers and data centers to gain insights into performance impacts. Their study explored 25 state-of-the-art applications and benchmarks across domains such as deep learning, graph analytics, and HPC, and evaluated interference effects across 625 co-running application pairs. The researchers analyzed runtime performance, resource usage, and bandwidth consumption to uncover insights about co-running interactions. They highlighted performance degradation patterns, identified contentious code regions, and proposed strategies to mitigate interference. This work offered guidelines for optimizing hardware utilization in modern computing systems. Advantages include improved throughput and resource allocation strategies, while limitations involve the complexity of characterizing diverse workloads and implementing effective mitigation techniques.

Jin Yang et al. [13] suggested a parallel computing framework for geodynamic numerical simulations optimized for the Tianhe HPC architecture. This framework addressed the inefficiencies of CitcomCu, a geodynamic simulation software, on large-scale heterogeneous computing systems. The authors optimized data partitioning to reduce communication overhead, improved iterative solution algorithms using the conjugate gradient (CG) method, and utilized the NEON Single Instruction Multiple Data (SIMD) instruction set for sparse matrix operations. These enhancements achieved a 3x performance increase and improved parallel efficiency by 16.22%. The framework’s main advantage lies in its ability to enhance computational performance and efficiency in large-scale simulations. The system’s dependence on specialized hardware, such as Central Processing Units (CPUs), Digital Signal Processors (DSPs), Double Data Rate (DDR) memory, Global Shared Memory (GSM), and High Bandwidth Shared Memory (HBSM), restricts its flexibility. This reliance may reduce its applicability across varied computing environments. Additionally, the use of the Message Passing Interface (MPI) for communication further ties the framework to specialized HPC platforms.

Johannes de Fine Licht et al. [14] presented a set of optimization transformations for High-Level Synthesis (HLS) code to improve performance and scalability in HPC applications. The method categorizes transformations into pipelining (loop-carried dependency resolution and pipelined loop fusion), scalability (vertical unrolling and dataflow architectures), and memory optimizations (memory striping and buffering). These techniques improve pipeline efficiency, increase parallelism, and optimize memory bandwidth utilization, enabling scalable hardware designs. The authors demonstrate the method’s effectiveness on HPC kernels, achieving up to 29,950x speedup. While the approach provides a systematic optimization framework, its implementation requires manual intervention and hardware-specific expertise.

Riccardo Cantini et al. [15] employed Block size ESTimation through Machine Learning (BLEST-ML), a methodology for estimating block sizes in data partitioning for HPC applications using supervised machine learning. BLESTML uses a series of tree-based classifiers trained on logs of past executions to predict optimal block sizes. These logs include information about algorithm types, dataset characteristics, and the details of the computing environment. The methodology helps minimize execution time and improve scalability by balancing the trade-off between task parallelism (dividing data into smaller parts for parallel execution) and the overhead of task management. BLEST-ML was implemented in the distributed computing library dislib, which is built on the PyCOMPSs (Programming COMPSs) framework for distributed task-based parallel programming. It was evaluated on various datasets and infrastructures, including MareNostrum 4 (MN4), a powerful supercomputer at the Barcelona Supercomputing Center. Experimental results show that BLEST-ML effectively determines suitable block sizes, outperforming traditional trial-and-error approaches and general-purpose autotuning frameworks (tools that adjust application parameters to improve performance). However, its reliance on extensive training data and specific execution environments may limit its applicability to unseen configurations.

Hang Song et al. [16] introduced a scalable parallel algorithm for solving compact banded linear systems efficiently. Their method uses Parallel Cyclic Reduction (PCR) to improve performance across heterogeneous architectures, including both distributed and shared-memory systems. The method, applicable to tridiagonal and wider banded matrices, minimizes memory usage and communication overhead by exploiting sparsity patterns and flexible data decomposition. Designed for high-resolution simulations of partial differential equations, it excels in applications like compressible turbulent flows, aeroacoustics, and electromagnetics. Implementation on the Summit supercomputer demonstrated exceptional scalability, particularly for 3D grid-based problems like the Taylor-Green vortex using a Navier-Stokes solver. The algorithm avoids costly”all-to-all” communication and adapts well to heterogeneous systems, achieving optimal performance with power-of-two partitions. By addressing computational and communication bottlenecks, this work enables efficient large-scale simulations on modern HPC platforms.

Existing approaches, including GAs and static scheduling methods, face limitations in scalability and adaptability, particularly in high-dimensional and heterogeneous environments. These methods often require substantial preprocessing or rely on fixed parameters that fail to adapt to dynamic workload variations. Limited data locality, higher computation cost, complexity in adapting to heterogeneous hardware platforms, challenges in workload adaptation, and high configuration complexity are the main drawbacks of the existing methods. In contrast, PSOALS leverages PSO’s collective intelligence to refine scheduling solutions iteratively, achieving superior convergence and adaptability. The innovative integration of wave angles as control parameters distinguishes this work by addressing dependencies and balancing workloads across processors effectively.

This study introduces a framework that combines mathematical rigor with practical implementation. It outperforms existing methods in execution time and computational efficiency. The proposed method, PSOALS, is a hybrid of PSO, GA, and the wavelength method for scheduling nested loops. It maintains a good balance between exploration and exploitation during optimization. A wave-angle scheduling mechanism is developed to optimize task execution order by balancing computational loads, minimizing communication delays, and maximizing parallelism. Unlike earlier approaches, PSOALS is flexible and adapts to diverse hardware architectures and workloads. This makes it suitable for modern HPC and IoT systems. Table 1 summarizes the related methods.

**Table 1:** Summary of the related research studies.

Reference	Proposed Method	Advantage	Disadvantage
Athanasaki et al. [9]	Four scheduling methods: mirror allocation, cyclic allocation, cluster allocation, and re-tiling scheduling.	Re-tiling scheduling performs better when tiles match SMPs; cluster allocation minimizes execution time.	Mirror allocation results in idle processors; cyclic allocation may lack data-locality optimization.
S. Parsa et al. [10]	Constraint GA for tiling in Cartesian spaces.	Reduces cache misses; handles higher-dimensional tiles flexibly.	Computational complexity and variability due to genetic operators.
A. Hajieskandar [11]	GA with bipartite chromosomes for loop scheduling.	Achieves superior solution quality and stability.	High computational cost; limited scalability for high-dimensional problems.
G. Galante [12]	Adaptive parallel programming with resource scalability and migration.	Improves performance and fault tolerance while supporting elastic scaling.	Complexity in adapting to heterogeneous hardware
H. Xu et al. [5]	Co-running interference analysis for throughput-oriented systems.	Enhances throughput; identifies and mitigates contentious code regions.	Characterizing workloads is complex
J. Yang et al. [13]	Parallel computing framework for geodynamic simulations on Tianhe HPC.	Improves performance and scalability significantly.	Limited generalizability due to dependence on specialized hardware.
J. de Fine Licht et al. [14]	Optimizing transformations for HLS codes in HPC.	Enhances pipeline efficiency and scalability.	Requires manual effort and hardware-specific expertise.
R. Cantini et al. [15]	BLEST-ML: ML-based block size estimation for HPC.	Balances task parallelism and overhead;outperforms traditional approaches.	Relies on extensive training data; limited for unseen configurations.
H. Song et al. [16]	PCR for compact banded linear systems.	Scales well to thousands of GPUs; minimizes memory and communication overhead.	Limited to specific problems; performance tied to power-of-two partitions.

## 4. Method

### 4.1. Problem statement

Efficient task scheduling is a critical challenge in computational systems, especially for applications involving nested loops with complex interdependencies. These loops are fundamental to many IoT applications, such as real-time monitoring, distributed data processing, and sensor network management. However, the sequential execution of nested loops often results in significant delays, underutilized processing resources, and increased communication overhead. These issues become even more pronounced in large-scale IoT systems, where the dynamic nature of workloads and the heterogeneity of devices further complicate scheduling. Existing scheduling methods, such as block-based, cyclic, or heuristic approaches, often lack the flexibility to adapt to dynamic dependencies and workload variations. These static methods typically focus on minimizing execution time without adequately addressing other critical factors, such as inter-task communication, energy efficiency, and real-time responsiveness. As IoT applications demand greater scalability and low-latency operation, the limitations of traditional scheduling strategies hinder their ability to handle modern IoT challenges, such as energy-constrained devices and heterogeneous resource capabilities. The need for a more adaptive, efficient, and dependency-aware scheduling framework has become increasingly urgent.

**Fig 6:**
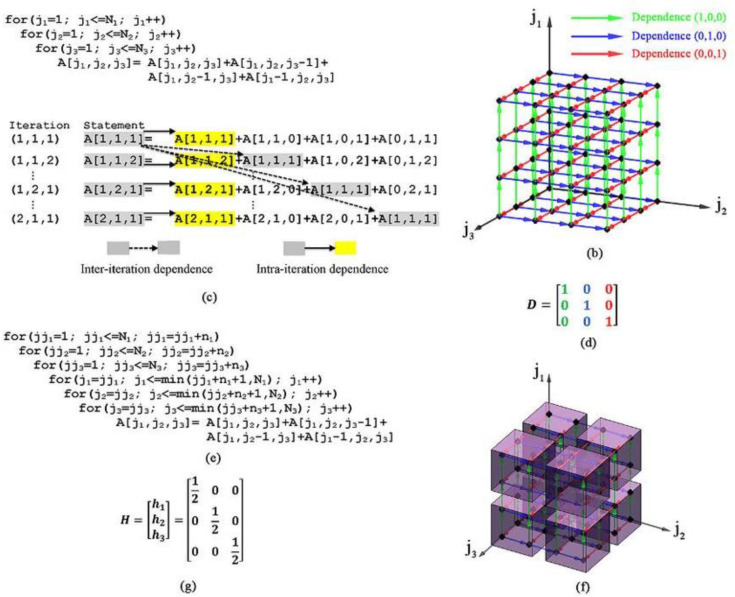
Nested loop dependencies and tiling strategies for optimized scheduling in IoT systems.

Fig 6 illustrates the complexity of managing nested loop dependencies, a key bottleneck in IoT task scheduling. Panel (b) in Fig 6 provides a 3D representation of task dependencies in a three-level nested loop, highlighting inter- and intra-iteration relationships. In Fig 6, the dependency matrix D (subgraph d) represents the dependency directions in the iteration space. The transformation matrix H (subgraph g in Fig 6) has one row for each dependency direction, meaning that each iteration depends on the previous iteration along each dimension. This represents a wavefront transformation, which is commonly used in wavefront parallelization. The transformation scales the iteration space to introduce wavefront execution patterns that respect dependencies while enabling parallel execution. The key idea is that the transformed indices define a new ordering, allowing execution to proceed in waves rather than strictly sequentially. Each transformed index represents a group of iterations that can be executed in parallel. The suggested method partitions the original iteration space into tiles using the tiling sizes n1, n2, and n3. The transformation matrix H in (subgraph g In Fig 6, the method helps define an execution wavefront; it maps each tile to a new wavefront schedule, enabling better parallel execution. The tiled iteration space in (subgraph f in Fig 6) shows the effect of wave-based scheduling, where each tile (block of iterations) is scheduled based on wavefront parallelization principles.

The tile size and wave angle (a, b) settings in the PSOALS algorithm are crucial parameters that directly influence performance and are typically adjusted experimentally based on workload characteristics and platform constraints; this parameter is application dependent and can be adjusted based on the input program. Tile size is often chosen by balancing parallelism, granularity, and communication overhead. Smaller tiles increase parallelism and scheduling flexibility but can cause excessive overhead due to communication. Larger tiles reduce overhead but may underutilize processors. Hence, the algorithm may either start with default tile sizes and refine them based on execution time. Wave angle parameters (a, b) define the diagonal direction of the scheduling wavefront, which affects how tiles are prioritized for execution. These values are tuned to respect data dependencies while minimizing idle processor time. In PSOALS, these settings can be adjusted during the fitness evaluation phase, where configurations leading to better load balance and shorter execution times are favored.

### 4.2. Particle swarm optimization

Algorithm 1 shows the pseudocode of the suggested PSOALS for loop parallelization. The PSOALS pseudocode outlines a hybrid optimization strategy for parallelizing nested loops in programs. The process begins with data dependence analysis, ensuring that iterations are tiled without violating logical execution order. These tiles represent sub-regions of the iteration space and serve as units of work to be scheduled across multiple processors. The scheduling problem is modeled using PSOALS, in which each particle represents a distinct mapping of tiles to processors. The particles’ fitness is evaluated based on key metrics such as makespan (total execution time), communication cost, and load balancing. This evaluation considers the wave-angle model, which introduces (a, b) parameters to influence the execution order and shape of the scheduling wavefront.

To enhance exploration and avoid premature convergence, the algorithm integrates GA techniques, such as mutation and gene swapping, into the PSO process. These operations introduce diversity into the swarm by randomly reassigning tasks, thereby allowing the algorithm to escape local optima. After iterating through a predefined number of generations and updating personal and global best solutions, the best particle is used to generate the final task schedule. This schedule respects all data dependencies and distributes the computation effectively across available resources using the wave-angle strategy. Finally, the optimized loop code is automatically generated for parallel execution, resulting in improved performance, scalability, and adaptability across heterogeneous IoT systems.

**Algorithm 1** **. Pseudocode of the suggested PSOALS**

**Input**: Nested loop structure in a source code

**Output**: Optimized and parallelized the source code

**BEGIN**

// *Phase 1: Loop Analysis and Tiling*

Perform data dependence analysis on the nested loops

Tile the iteration space into blocks based on loop bounds and dependencies

**Initialize** wave-angle parameters (a, b) for scheduling geometry

*// Phase 2: Particle Initialization (PSOALS)*

Initialize a swarm of particles:

**Each** particle represents a possible scheduling of tiles

Encode particle position as processor assignment for each tile

**Initialize** velocities randomly

**FOR** each particle

Evaluate fitness based on:

Communication cost

Load balancing

Makespan

Dependency constraints

**END FOR**

*// Phase 3: PSOALS Iteration*

**WHILE** termination condition not met (max iterations)

**FOR each** particle

**Update** velocity using PSO formula:

v_new = ω * v_old + c1 * r1 * (p_best – x) + c2 * r2 * (g_best – x)

Update position: x = x + v_new

Enforce boundary and validity constraints

Apply mutation with low probability:

Bit-flip (change processor)

Swap two assignments (GA operation)

Evaluate fitness of the new position

Update personal best (p_best) and global best (g_best)

**END FOR**

**END WHILE**

*// Phase 4: Final Scheduling*

Apply wave-angle scheduling using the best particle (g_best)

Schedule tiles in wavefront manner based on (a, b)

Respect tile dependencies and minimize inter-processor communication

Generate optimized parallel loop code from the best schedule

**END**

The PSO algorithm is an innovative optimization technique that operates on a population [17]. In the PSO algorithm, each problem solution is represented as a particle in the search space. Each particle has a fitness value, which is calculated by the fitness function of the problem. The particle closer to the answer has a higher fitness value. This algorithm is a swarm-based search method that starts with a group of random solutions (particles) and iteratively updates their positions to find the optimal solution in the problem space. Two representations define each particle $X_{id}$ and $V_{id}$, where $X_{id}$ represents the position of the *d*-th dimension of *i*-th particle and $V_{id}$ represents the speed of *d*-th dimension of the *i*-th particle. In each stage, the location of each particle is updated according to the two best values:

- $p_{best}$ **(personal best):** It is the best experience that the particle itself has gained so far,
- $g_{best}$ **(global best):** It is the best experience obtained among all the particles.

**Fig 7:**
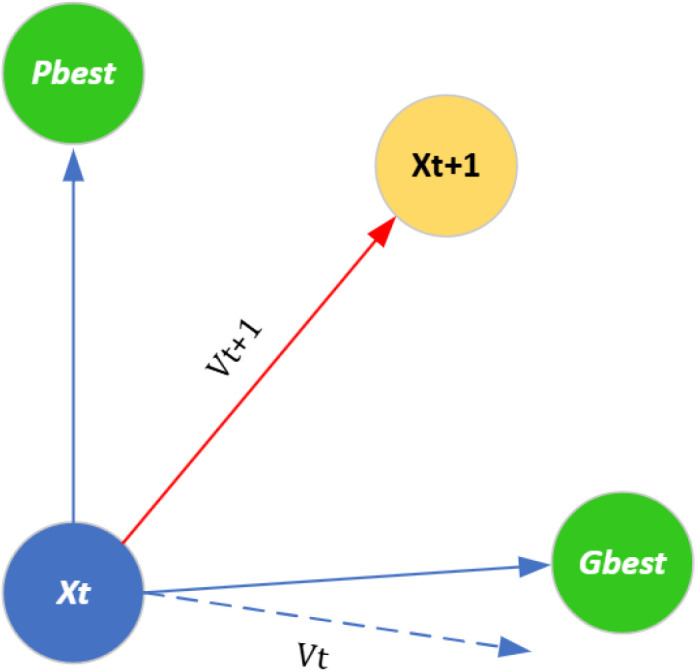
Movement of each particle in the PSO.


Fig 7 displays how the particle moves until it reaches the target. In each iteration, the algorithm updates the new position of the particle based on Eq. (1) after finding the two values of $p_{best}$ and $g_{best}$. In Eq. (1), V_*id*_(*t* + 1) represents the updated velocity of particle *i* and *V*_*id*_ (*t*) is the current velocity.

$$V_{id}(t\;+\;\text{1})=\;w\times V_{id}(t)+\;C_{\text{1}}\times rand\times \left(p_{{best}_{id}}(t)-{x\;}_{id}(t)\right)+C_{\text{2}}\times rand\times \left(g_{{best}_{d}}(t)-{x\;}_{id}(t)\right),$$ (1)

where *w* is the inertia coefficient that is between (0*,*1), *C*_1_ and *C*_2_ are learning or acceleration coefficients that are chosen in the range, which are equal to 2 in most cases, and the rand is a random number in the range [0*,*1]. After obtaining *v*, it is used in Eq. (2) to obtain the new location of the particle:


$${x\;}_{id}(t+\text{1})={x\;}_{id}(t)+V_{id}(t+\text{1})\;(i=\text{1},\text{2},...,N;\;d=\text{1},\text{2},...,D).$$ (2)

### 4.3. Proposed PSOALS

The suggested PSOALS operates in a discrete search space, in which each task or loop iteration must be assigned to a specific processing unit. However, the native PSO algorithm generates real-valued position vectors, which are not directly applicable to discrete assignment problems. To address this, the continuous positions produced by PSOALS are discretized using the *round()* function. This function converts each real-valued position into the nearest integer, effectively mapping each loop iteration or task to a valid processor ID. It ensures that each task is assigned to a valid resource while preserving the directional guidance of the PSOALS velocity updates. Despite its simplicity, this discretization approach has proven effective in our scheduling context, enabling PSOALS to explore the solution space efficiently and produce high-quality parallelization across heterogeneous IoT platforms.

In the movement stage, PSOALS combines PSO and GA to provide a more powerful and effective search mechanism. The heuristic algorithms may not only guarantee an optimal solution, but they can also achieve good results in the nested loops. Unlike traditional techniques that rely on a fixed configuration, PSOALS incorporates dynamic wave-angle adjustments and evolutionary optimization to minimize execution time and enhance resource utilization. The generation and analysis of all possible wave angles enable a refined approach to scheduling, offering significant advantages in scenarios involving complex dependencies and large-scale parallel computing systems. The PSOALS method integrates PSO with wave-angle-based scheduling to optimize the execution of nested loop tasks in IoT environments. The proposed method systematically addresses challenges such as dependency resolution, workload balancing, and resource utilization across distributed IoT devices. Below, we present the methodology in a detailed 10-step process.

#### Step 1: Define the iteration space.

The method begins by modeling nested loops as a two-dimensional iteration space l, where each point (i, j) represents a computational task. In Eq. (3), the bounds N and M are determined by loop ranges in the IoT application. Dependencies between tasks are represented as directed edges in a dependency graph D, ensuring task execution adheres to these constraints.


I={(i,j)|0≤i<N, 0≤j<M}. (3)

The dependency graph is defined by Eq. (4). These definitions establish a mathematical framework for optimizing execution order guided by the dependency constraints.


$$D=\left\{\left((i,j),\left(i^{\prime },j^{\prime }\right)\right)\;|\;i^{\prime }\leq i\text{ and }j^{\prime }<j\right\}.$$ (4)

#### Step 2: Analyze dependencies and identify critical paths.

The execution order of tasks in IoT systems depends heavily on resolving dependencies. These dependencies can span multiple iterations and loops, significantly impacting performance. A critical path is the longest chain of dependent tasks in the iteration space, representing the minimum execution time required for the entire task set. According to Eq. (5), the critical paths are identified by summing the weights (execution costs) of tasks along each dependency chain:


$$Critical\;Path={\text{max}}_{(i,j)\in l}\;\sum_{k=\text{1}}^{K}W_{k},$$ (5)

where $W_{k}\;$ represents the workload of tile *k* (or *k*-th task). Here, $\sum_{k=\text{1}}^{K}W_{k}$ indicates the total workload along a given dependency path. Moreover, (i,j) shows pairs of tiles connected in the dependency graph and tile i must be executed before tile j, and finally, K indicates the number of tiles (tasks). This analysis highlights bottlenecks and helps prioritize tasks to improve overall system performance. In IoT systems, where some devices may have limited computational resources, understanding critical paths ensures effective load distribution and minimizes delays.

#### Step 3: Tile the iteration space.

To enhance parallelism and scalability, the iteration space is divided into smaller tiles, each containing a group of tasks. Tiling allows the execution of multiple tiles in parallel, provided there are no inter-tile dependencies. Each tile $T_{kl}$ is defined by Eq. (6):


$$T_{kl}=\{(i,\;j)|k\times t\;\leq i<(k+\text{1})\times t,\;l\times t\;\leq j<(l+\text{1})\times t\},$$ (6)

where k and l index the tiles, and t is the tile size. Dependencies between tiles, $D_{T}$, are determined by Eq. (7):


$$D_{T}=\left\{\left(T_{kl},\;T_{k^{\prime }l^{\prime }}\right)|T_{kl}\text{ depends on}{\;T}_{k^{\prime }l^{\prime }}\right\}.\;$$ (7)

**Fig 8:**
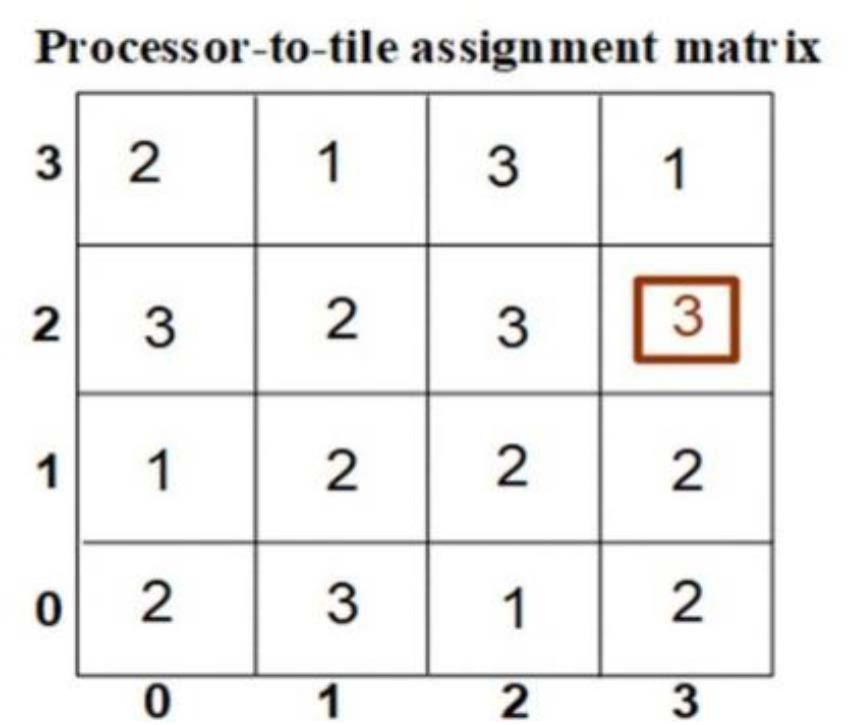
Processor-to-tile assignment matrix.

In IoT systems, tiling reduces scheduling complexity by aggregating tasks into manageable units, while dependency analysis ensures correctness in execution. Fig 8 depicts an example of processor-to-tile assignments in the system.

#### Step 4: Introduce wave angle scheduling.

Wave angle scheduling defines the execution order of tiles by introducing wavefronts that respect dependency constraints. Wave angle scheduling is a technique used in loop tiling to define the execution order of tiles while respecting dependency constraints. The wave number wn for each tile is calculated by Eq. (8):


wn=a × k + b × l,  (8)

where a and b control the slope of the wavefront, and |a|, |b|≤m limit the range of wave angles. *k* and l are tile indices; *a* and *b* are coefficients that control the scheduling order. The wave angle θ is defined in Eq. (9):


$$tan\;\theta =\frac{b}{a}.$$ (9)

**Fig 9:**
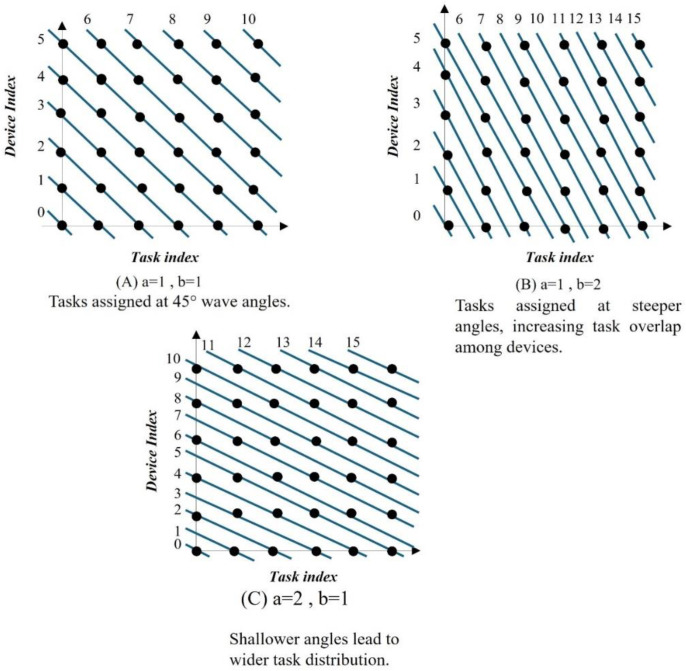
Wavefront scheduling with different wave angles. (A) Balanced task distribution, (B) steeper angles with task overlap, (C) shallow angles with a wider distribution.

Tiles with the same wn can be executed in parallel, while wavefronts are processed sequentially. This scheduling method optimizes parallelism by ensuring that all independent tasks are executed simultaneously, minimizing idle times for IoT devices. Fig 9 demonstrates the impact of different wave angles on task distribution and overlap. In Fig 9(A), a=1,b=1 is generally a good calibration as it provides a balanced workload with minimal synchronization issues. In Fig 9(B), a=1, b=2 could be problematic due to higher contention between processors. In Fig 9(C), a=2, b=1 may work well for independent tasks but can cause underutilization if tasks are not evenly distributed. Generally, if dependencies between tasks are minimal, option (A) is the best choice for efficient parallel execution.

#### Step 5: Encode tiles as particles.

Each tile is represented as a particle in the PSO framework, enabling systematic optimization of task assignments. As displayed in Eq. (10), the particle-matrix M encodes processor assignments for tasks within tiles. In Eq. (10), $M_{kl}$ is an element in the population matrix M; it shows the processor assignment for tasks within the tile ($T_{kl}$). Each tile (task) contains multiple tasks of (i, j) that must be assigned to a processor. Furthermore, $P_{ij}$ represents the processor assigned to task (i, j). As formulated by Eq. (11), a swarm S of s particles represent multiple candidate schedules.


$$M_{kl}=\;P_{ij}\;\forall i,\;j\in T_{kl},\;$$ (10)

$$S=\left\{M^{\left(\text{1}\right)},M^{\left(\text{2}\right)},...\;,M^{\left(s\right)}\right\}.$$ (11)

Encoding tiles as particles allows the PSO algorithm to evaluate and improve task scheduling systematically, taking into account dependency constraints and resource limitations.

#### Step 6: Define the fitness function.

The fitness function evaluates the efficiency of task assignments in IoT environments. The objective is to minimize the makespan, which is the total execution time while considering both computational and communication costs inherent to IoT systems. Computation Time represents the time required to compute the operations within the tile. Communication overhead captures the overhead due to data exchange between different tiles or processing units. Communication overhead arises from data dependencies, synchronization delays, and memory access overhead. Synchronization overhead in wavefront tiling (where some tiles must wait for dependencies) contributes to communication overhead. For a given particle (schedule) M, the fitness function is defined by Eq. (12). In an IoT system, where tasks (tiles) are distributed across multiple processors $P_{i}$, the cost of executing a tile $T_{kl}$ is determined using Eq. (13). Therefore, $P_{i}$ indicates processor *i* in the IoT system. Moreover, $T_{comp}$ indicates the time required for processor Pi to execute the computations within the tile $T_{kl}$, which depends on processor speed, workload, and complexity of the task. Hence, $T_{comp}$ indicates the time taken to transfer data between tiles or processors; this time is affected by network latency, bandwidth, and data dependencies.


$$f(m)=\frac{\text{1}}{T_{total}};\;T_{total}={\text{max}}_{P_{i}}\;\left(\sum_{Tkl\in P_{i}}C(Tkl)\;\right),$$ (12)

$$C\;\left(T_{kl}\right)=\;T_{comp}\left(T_{kl}\right)+T_{comm}\left(T_{kl}\right).$$ (13)

Eq. (14) is used to calculate $T_{comp}$ and $T_{comm}$. In this equation, $T_{comp}(T_{kl})$ is the time required to compute the tasks in $T_{kl}$, and $T_{comm}(T_{kl})$ represents the time to transfer data between dependent tiles across processors or IoT nodes. The hardware capabilities and network bandwidth influence these costs in the IoT environment. To adapt to heterogeneous IoT nodes, In Eq. (14), where $w_{ij}$ is the computational weight, $d_{ij}$ is the data transfer size and $\beta_{ij}$ is the network bandwidth. Moreover,$\alpha_{ij}$ indicates the processing speed of the assigned processor for task (i,j). The computational workload $w_{ij}$ of task (i,j) represents the amount of computation required to execute that task. It is typically measured in terms of floating-point operations (FLOPs), CPU cycles, execution time, or instructions. This fitness function ensures task assignments are optimized for IoT-specific constraints. We incorporate a weighting factor $\alpha_{ij}$ based on the processing capacity of node i and the communication link quality to node j.


$$T_{comp}\left(T_{kl}\right)=\sum_{(i,j)\in T_{kl}\;}\frac{w_{ij}}{\alpha_{ij}}\;,\;T_{comm}\left(T_{kl}\right)=\sum_{(i,j)\in T_{kl}}\frac{d_{ij}}{\beta_{ij}}.$$ (14)

#### Step 7: Update particle velocities and positions.

The PSO algorithm refines task schedules by iteratively updating particle velocities and positions. Each particle represents a candidate schedule for the IoT environment. The updates consider computational dependencies and resource constraints unique to IoT systems. The velocity update is calculated by Eq. (15):


$$v_{ij}(t+\text{1})=w\;\times v_{ij}(t)+c_{\text{1}}\times r_{\text{1}}\times \;\left(p_{{best}_{ij}}-x_{ij}(t)\right)+c_{\text{2}}\times \;r_{\text{2}\;}\times \left(g_{{best}_{ij}}-x_{ij}(t)\right),$$ (15)

where w is the inertia weight, $c_{\text{1}}$ and $c_{\text{2}}$ are learning coefficients, and $r_{\text{1}}$, $r_{\text{2}\;}$ are random numbers within [0, 1]. The position update is calculated by Eq. (16):


$$x_{ij}(t+\text{1})=x_{ij}(t)+v_{ij}(t+\text{1}).$$ (16)

To enforce IoT-specific constraints, positions $x_{ij}$ are clipped to valid ranges reflecting the available processors and bandwidth limitations by means of Eq. (17). A dependency-aware adjustment ensures that tiles with unresolved dependencies do not move to invalid positions. In Eq. (17), $x_{dep,ij}$ represents the maximum allowable position based on dependencies:


$$x_{ij}(t+\text{1})\leftarrow \text{min }\left(x_{ij}(t+\text{1}),x_{dep,ij}\right).$$ (17)

#### Step 8: Apply crossover and mutation for diversity.

In this study, a hybrid variant of the PSO was used for the loop parallelization problem. In the developed hybrid PSO, crossover, and mutation, two well-known GA operators, are utilized to enhance the PSO performance. GA is considered as a popular population-based evolutionary algorithm used to solve optimization problems. Crossover and mutation play an important role in improving new chromosomes. In the suggested hybrid PSO, a 2-point crossover operator has been performed on the $p_{best}$ and $g_{best}$ after performing the PSO classic movement operators (Eqs. (1) and (2)). The newly created offspring (or particle) consists of two parts. One represents the best position of the particle with the best performance in the group and the other captures the best position found by the local best. In the experiments, the 2-point crossover gave better results than the 1-point crossover. Furthermore, the one-pint mutation operator has been applied to $p_{best}$and $g_{best}\;$particles. The mutation operator serves two main purposes such as avoiding the local optima and reducing the probability of getting stuck in suboptimal solutions. If the PSO movement (Eqs. (1) and (2)) and crossover fail to improve particles beyond $p_{best}$and $g_{best}$, the mutation operator is applied to $g_{best}$. The best solution that emerges at the end of all iterations is $g_{best}$. If the mutation operator fails to improve $g_{best}$, it is applied to $p_{best}$. If there is still no improvement, the mutation process extends to *X*_*new*_. Mutation operations in PSOALS are tailored to address the heterogeneity and dynamic nature of IoT environments. Two mutation strategies are employed:

1 **Resource-Aware Bit Flipping:** Processor assignments for a randomly selected task (i, j) are altered within the range of available IoT nodes using Eq. (18):

$$M_{ij}\leftarrow rand\;\left(\text{1},P_{avail}\right).$$ (18)

2 **Dependency-Preserving Swapping:** Tasks in two tiles $T_{kl}$ and ${T^{\prime }}_{k^{\prime }l^{\prime }}$ are swapped only if the resulting schedule preserves dependencies and improves the makespan based on Eq. (19):

$$\text{if }f\left(M^{\prime }\right)>\;f(M),\text{ then }M_{ij}\;↔\;M.$$ (19)

**Fig 10:**
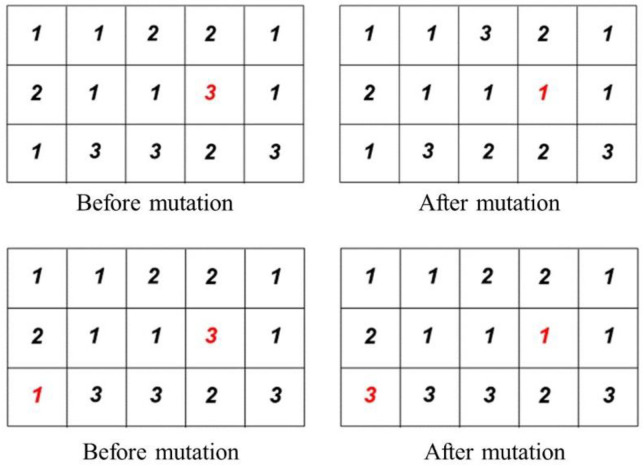
Bit flipping changes and Gene swapping as mutation operator in the suggested PSOALS.

These targeted mutations prevent premature convergence while ensuring schedules remain feasible within IoT-specific constraints. Fig 10 illustrates the mutation operation applied to particles in the PSOALS algorithm, where each particle represents a task assigned to a processor. The mutation process introduces variation by modifying task-to-processor assignments, which helps the algorithm explore new scheduling configurations.

#### Step 9: Evaluate convergence.

Convergence in PSOALS is determined by monitoring the stability of the makespan across iterations. An adaptive threshold, ∈(t), is employed to account for system variability in dynamic IoT environments. The convergence condition is defined by Eq. (20):


$$||\;f\;(\overset{\left(t\right)}{\underset{best}{g}}-f(\overset{\left(t-\text{1}\right)}{\underset{best}{g}})||<\in (t),$$ (20)

where ∈(t) decreases as the algorithm progresses, encouraging exploration during initial iterations and refining solutions in later stages. This dynamic threshold is periodically adjusted based on real-time resource metrics, such as processor load and communication delays, ensuring practical relevance and robustness.

#### Step 10: Deploy optimized schedule to IoT nodes.

The final schedule $g_{best}$ is translated into actionable instructions for the IoT nodes. Each task is assigned to its respective processor with a clear execution order that respects both intra-tile and inter-tile dependencies. The deployment process minimizes latency and balances resource utilization by prioritizing tasks with high criticality. The total execution time for the schedule is calculated using Eq. (21):


$$T_{total}=\sum {\mathrm{\backslash nolimits}}_{T_{kl}\in \tau }(T_{comp}(T_{kl})+T_{comm}(T_{kl})).$$ (21)

Before deployment, a validation step ensures that the schedule meets all dependency constraints. Real-time monitoring feedback is integrated to dynamically adjust task priorities in case of unexpected IoT node failures or communication delays. The optimized schedule is disseminated to IoT devices through a hierarchical control structure, with a master node overseeing execution progress and handling runtime adjustments.

## 5. Experiments

### 5.1. Platform

**Fig 11:**
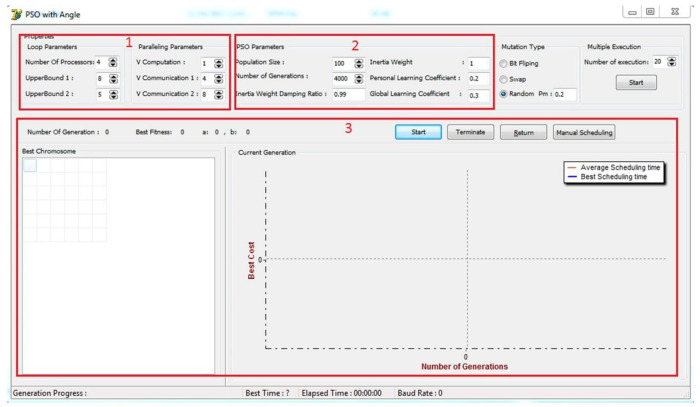
Main interface of the implemented software platform.

In this section, firstly, the simulated software that is used to test the presented algorithm is introduced, and then the results obtained from the implementation of the developed method and a group of existing algorithms are displayed. Algorithms used to compare with the proposed method are horizontal block (BlockH), vertical block (BlockV), horizontal cyclic (CyclicH), vertical cyclic (CyclicV), genetics or Genetic Algorithm-based Loop Scheduling (GALS), and PSO without the change of the wave angle. The parameters for performing each test are given in the relevant section. Algorithms have been compared from four points of view: convergence to the optimal solution, stability of algorithms, reliability, and the quality of their generated solutions. In this section, the simulation software implemented in the Delphi 10 environment is explained. As seen in Fig 11, this interface consists of the following main parts: loop parameters, parameters of the PSO algorithm, and program output. The parameters that determine the dimensions of the loops and the parallel system are explained below. It should be noted that the lower bound of the loops is considered to be zero by default.

- NP: Number of processors (number of processors in the system),
- UB1: UpperBound1 (upper bound of the outer loop),
- UB2: UpperBound2 (upper bound of the inner loop),
- Vcomp: $V_{\text{computation}}$ (volume of intra-processor computations),
- Vcomm1: $V_{\text{computation1}}$ (volume of communication between processors in the direction of the first dimension),
- Vcomm2: $V_{\text{computation2}}$ (volume of communication between processors in the direction of the second dimension). It should be noted that the lower bound of the loops is considered to be zero by default.

As shown in Fig 11, the implemented simulation platform offers a comprehensive and configurable environment under various parallel processing scenarios. The implemented platform has been carefully designed to emulate realistic execution environments by allowing users to configure algorithmic parameters, monitor execution, and visualize scheduling performance. All key hardware and software platform features have been taken into account in the design of the simulation environment. The platform allows experiments to be conducted under different hardware configurations, including variations in the number of processing cores, computational power, and communication overhead. These capabilities enable researchers to assess the scheduling efficiency of PSOALS under both homogeneous and heterogeneous execution conditions that are common in real IoT and embedded systems.

The simulation environment consists of three major configurable components. The first handles hardware-level platform parameters, where users can define the number of processors and adjust parameters such as *V* Computation and *V* Communication (representing the cost of computation and inter-processor communication, respectively). These factors play a critical role in real-time systems and have been directly integrated into the scheduling logic, effectively simulating the constraints and dynamics of real hardware platforms. The second component focuses on the algorithmic configuration of PSOALS. It enables precise control over parameters such as population size, number of generations, inertia weight, and learning coefficients. This allows for extensive experimentation to explore the algorithm’s adaptability and convergence behavior in different problem spaces. Furthermore, genetic mutation strategies are incorporated to enhance diversity in the search process and reduce the likelihood of premature convergence, ensuring robustness across multiple runs.

The third component of the platform is dedicated to execution control and visualization. Real-time output metrics such as the best chromosome, fitness values, and convergence trends are displayed dynamically. This not only enhances the interpretability of results but also allows researchers to monitor algorithmic behavior as it evolves over time. Indicators like best time, elapsed time, and generation progress further support in-depth analysis. The flexibility and validity of the developed platform offer a powerful and extensible alternative for initial validation. The simulation environment was deliberately designed to support the simulation of dynamic scheduling behavior in multi-processor and resource-constrained systems, which closely resembles the conditions found in real-world IoT deployments. This ensures that the results generated are meaningful, reproducible, and relevant to a wide range of practical scenarios.

The values of the configuration parameters have been shown by Table 2. The configuration parameters of the PSOALS algorithm are tuned to ensure an effective balance between exploration and exploitation. A population size of 80 provides sufficient diversity for the search space while maintaining computational efficiency. The algorithm is run for 2000 generations, allowing it to iteratively refine solutions toward optimality. The inertia weight is set to 1, with a damping ratio of 0.99, gradually decreasing the momentum to transition from global exploration in early stages to local exploitation in later iterations. The personal and global learning coefficients are set to 0.2 and 0.3 respectively. Mutation is applied using a random probability (Pm) of 0.2 to introduce controlled diversity and avoid local optima. Finally, the algorithm is executed 20 times to ensure consistency and reliability of the results through multiple trials. These parameter settings are selected specifically to enhance the performance of loop parallelization in dynamic and heterogeneous IoT environments.

**Table 2:** Configuration Parameters of the PSOALS.

Parameter	Value	Description
Population Size	80	Number of particles in the swarm
Number of Generations	2000	Iteration limit for the algorithm
Inertia Weight	1	Controls exploration vs. exploitation
Inertia Weight Damping Ratio	0.99	Decreases the inertia weight over generations
Personal Learning Coefficient (c1)	0.2	Influence of personal best position
Global Learning Coefficient (c2)	0.3	Influence of the global best position
Mutation Type	Random Pm	Type of mutation applied
Mutation Probability (Pm)	0.2	Probability of applying a mutation
Number of Executions	20	Repeated runs for result averaging or comparison

### 5.2. Evaluation of the proposed algorithm

This section analyzes the convergence, stability, and reliability of the suggested algorithm, PSOALS. Mathematical formulations, experimental results, and visual illustrations support each evaluation metric.

#### 5.2.1. Convergence analysis.

**Fig 12:**
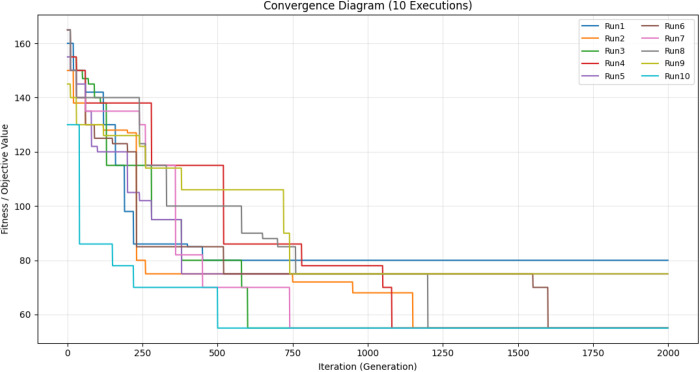
Convergence of the proposed algorithm for the problem with dimensions 4 × 4 (Vcomputation = 1, Vcommunication = 4).


To evaluate the convergence of the proposed PSOALS algorithm, the fitness value is computed at each generation. Let $fg_{\text{best }}$ denote the best fitness value at generation g, and f(g) represent the average fitness value at the same generation. The convergence is mathematically expressed as $\;f\overset{\left(t\right)}{\underset{best}{g}}$ denoting the best fitness value at generation g, and $\overset{―}{f}(g)\;$ represents the average fitness value at the same generation. The convergence is mathematically expressed by Eq. (22) and using Eq. (23), where $\overset{\left(g\right)}{\underset{i}{f}}\;$ is the fitness value of the i-th particle at generation g, and np is the number of particles. Fig 12 illustrates the convergence of the developed algorithm for a 4 × 4 task scheduling problem on three processors over 2000 generations.

$$\begin{array}{c} f\overset{\left(t\right)}{\underset{best}{g}}=\text{ min }\{\overset{\;(g)}{\underset{i}{f}}|\;i\;=\;\text{1},\text{2},...,\;n_{p}\}, \end{array}$$ (22)

$$\begin{array}{c} \overset{―}{f}(g)=\frac{\text{1}}{n_{p}}\sum \overset{n_{p}}{\underset{i=1}{\mathrm{\backslash nolimits}}}\overset{\;(g)}{\underset{i}{f}}. \end{array}$$ (23)

As shown in Fig 12, the convergence plot illustrates how the proposed optimization strategy performs across 10 independent runs, each with 2000 iterations. The vertical axis depicts the fitness (objective) value, and the horizontal axis showsthe iteration (generation) number. At the start of the search, all runs begin with relatively high fitness values, indicating that the solutions were randomly initialized. In the first 200–400 generations, all executions exhibit a rapid decline in fitness. This suggests that the approach can rapidly explore a large portion of the search space and identify promising regions. The convergence curves become flatter as the iterations proceed. This shows that the process is moving from exploration to exploitation. Some runs stabilize their fitness values between 600 and 1200 iterations, whereas others continue to improve into later generations. This behavior shows that the algorithm is stochastic and can get out of local optima in more than one run. The fitness values remain largely unchanged in the later phases of the optimization process, indicating that the algorithm has found near-optimal or optimal solutions. There are minor differences across runs, but all yield competitive final fitness values, indicating that the proposed strategy is robust and stable. The convergence diagram shows that the proposed algorithm converges quickly, performs well across multiple runs, and strikes a good balance between exploration and exploitation. This makes it a suitable choice for addressing challenging optimization problems.

#### 5.2.2. Stability analysis.

To evaluate stability, the algorithm is executed multiple times under identical conditions, and the standard deviation ($\sigma_{f}$) of the best fitness values across runs is calculated using Eq. (24). Here, $\overset{\left(i\right)}{\underset{best}{f}}$ is the best fitness value for the i-th execution, $f_{best}$ is the mean best fitness value, and m is the total number of executions.


$$\sigma_{f}=\sqrt{\frac{\text{1}}{m}\sum \overset{m}{\underset{i=\text{1}}{\mathrm{\backslash nolimits}}}(\overset{\left(i\right)}{\underset{best}{f}}-\overset{―}{\;f_{best}}}{)}^{\text{2}}.$$ (24)

**Fig 13:**
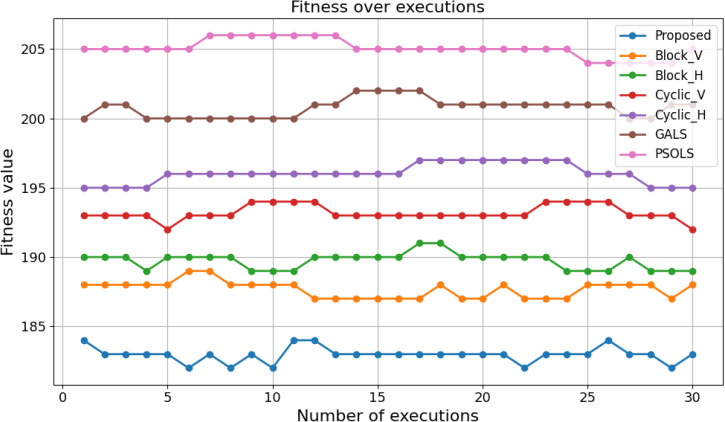
Stability of the proposed algorithm for the problem with dimension 5 × 5, (Vcomputation = 8, Vcommunication = 4) over 30 independent executions.

For a 4 × 4 scheduling problem with Vcomputation = 8 and Vcommunication = 4, the calculated fitness is shown in Fig 13. The fitness values consistently fall within the range 183 ± 1, and the standard deviation is minimal. Only minor deviations are observed, further validating the algorithm’s consistency and robustness. The developed method demonstrates superior stability compared to alternative approaches. As represented in Fig 13, our method consistently maintains the lowest standard deviation across all runs, while other methods, such as GALS and PSOLS, exhibit higher variability. The stability of PSOALS is attributed to its well-balanced exploration and exploitation mechanisms and its ability to avoid being trapped in suboptimal regions of the search space. This high stability is critical in practical applications, especially in task scheduling problems, where reproducibility and reliability are paramount. Stable performance ensures that the algorithm delivers consistent results regardless of variations introduced by stochastic elements or random initializations. Additionally, the ability to consistently converge to near-optimal solutions highlights the robustness of the proposed method, making it a reliable choice for real-world IoT scheduling scenarios.

Table 3 presents the standard deviations for the proposed method and the comparison methods. The standard deviation indicates the degree of variability across multiple executions; lower values indicate more consistent performance. The proposed method exhibits a low standard deviation (0.556053), indicating stable performance and minimal variation across runs. Its stability is superior to that of Block_V, Block_H, GALS, and PSOLS, and it is very close to that of the best-performing method, Cyclic_V. In contrast, Cyclic_H and GALS show higher standard deviation values, which indicate larger fluctuations during execution. Overall, the results show that the proposed method delivers reliable, consistent performance while maintaining competitive stability relative to other methods.

**Table 3:** The standard deviation of the different methods.

	Proposed Method	Block_V	Block_H	Cyclic_V	Cyclic_H	GALS	PSOLS
Standard Deviation	0.556053	0.606478	0.583292	0.550861	0.718395	0.678911	0.639684

#### 5.2.3. Reliability.

**Fig 14:**
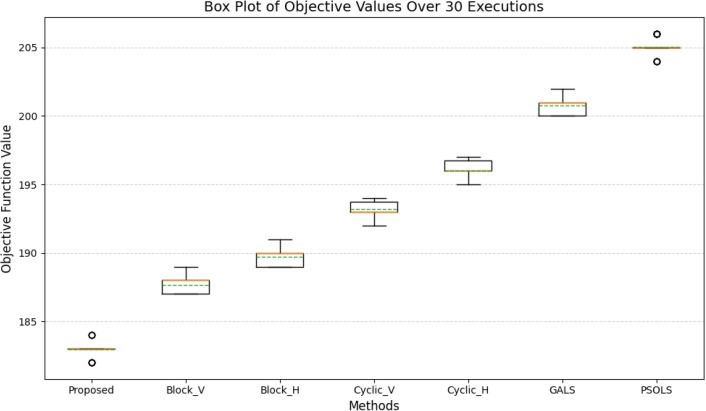
Box plot of the proposed algorithm compared with other methods for the problem with dimensions 5 × 5 (Vcomputation = 8, Vcommunication = 4).

Reliability is evaluated using two metrics: the success rate ($P_{success}$) and the average error ($E_{avg}$). The success rate is defined by Eq. (25), where $n_{success}$ is the number of executions that produced the optimal solution, and *m* is the total number of executions. Furthermore, the average error is computed using Eq. (26). In this study, the optimal solution is defined as the best fitness value obtained across 30 independent runs of the proposed method. Each execution is performed under identical parameter settings (population size, number of iterations, and algorithm configuration) but with different random initializations. The optimal value corresponds to the minimum fitness observed among all executions and serves as a reference point for evaluating reliability and success rate. The average error is computed using Eq. (26) as the mean of the differences between the best solution obtained in each execution and the experimentally observed optimal value. It is important to note that, because heuristic optimization algorithms are stochastic, global optimality cannot be guaranteed in theory.


$$P_{success}=\frac{n_{success}}{m}\times \text{1}\text{0}\text{0},$$ (25)

$$E_{avg}=\frac{\sum_{i=\text{1}}^{m}\left|\overset{\left(i\right)}{\underset{Obtained}{\;f}}-f_{optimal}\right|}{m},$$ (26)

where f (i) is the solution obtained in the i-th execution, and foptimal is the optimal solution. Fig 14 presents a box plot of the obtained fitness values from 30 independent runs for the proposed method and competing algorithms. The proposed method exhibits the lowest median and a very narrow interquartile range, indicating that its results are consistently close to the optimal solution across repeated runs. The compact distribution and short whiskers reflect low variability, while the absence of notable outliers demonstrates strong robustness against random initialization and stochastic effects. These characteristics confirm that the proposed approach delivers stable and repeatable performance. In contrast, the baseline methods (Block_V, Block_H, Cyclic_V, Cyclic_H, GALS, and PSOLS) exhibit wider interquartile ranges and higher medians, indicating greater dispersion and reduced consistency. The longer whiskers and presence of higher-value spreads indicate sensitivity to execution conditions and less reliable convergence behavior. Overall, the box plot analysis indicates that the proposed method not only achieves higher solution quality but also maintains superior stability and reliability across all executions compared with competing approaches.

### 5.3. Benchmarking the method

To evaluate the effectiveness of the proposed PSOALS algorithm for loop parallelization, a diverse set of loop workloads was utilized. Table 4 presents the workload specifications used to evaluate the proposed method. These workloads vary in loop dimensions and processor counts, allowing for comprehensive testing across different parallel execution scenarios. The loop nests range from smaller grids (4 × 6 and 6 × 6) to larger ones (10 × 10), and the number of processors used spans from 2 to 4. This variation ensures the algorithm’s performance is tested under both moderate and more computationally intensive conditions, reflecting realistic parallel computing environments.

**Table 4:** Workload used to evaluate the PSOALS method.

No.	Loop Dimensions	Number of Processors
1	6 × 6	2
2	4 × 6	2
3	8 × 4	3
4	10 × 6	3
5	10 × 6	3
6	9 × 7	4
7	10 × 10	3
8	9 × 4	3

#### 5.3.1. Benchmariking in 6 × 6 loop dimensions and 2 processors.

The results in Table 5 demonstrate the performance of the developed method compared to existing algorithms for a 6 × 6 task scheduling problem executed on two processors. The results highlight the efficiency and adaptability of the proposed method under conditions where the computation volume ($V_{comp}$) is significantly higher than the communication volumes ($V_{com\text{1}}$ and $V_{com\text{2}}$). PSOALS achieved the fastest execution time of 218, outperforming all comparison methods. Block-based methods (Block V and Block H) had significantly longer execution times of 274, while cyclic scheduling methods (Cyclic V and Cyclic H) demonstrated slightly better results with 228. GALS and PSOLS, which lack the wave-angle scheduling of PSOALS, achieved 220.

**Table 5:** Problem with dimensions 6 × 6 with 2 processors.

Algorithm inputs		Algorithm execution time						
		Previous methods						
Dimensions of the two-level loop		Method	Block V	Block H	Cyclic V	Cyclic H	GALS	PSOLS
UB1	6	Execution time	274	274	228	228	220	220
UB2	6	**Proposed method**						
Parallel system		Execution number	PSOALS	Angle coefficient		Result		
NP	2			a	b			
Vcomp	10	1	218	1	3	Best time	a	b
Vcom1	1	2	218	3	1	218	3	1
Vcom2	1	3	218	3	1	Average	218.2	
Number of iterations	4	218	3	1				
NG	2000	5	219	3	1	improve		

Our method consistently produced the best execution time across multiple executions. The results indicate that PSOALS is robust to variations in initial conditions and stochastic elements, maintaining low variability compared to other methods. Static block-based methods (Block V and Block H) exhibited higher variability due to their fixed partitions, which do not adapt to dynamic dependencies. The use of wave angle scheduling, with angle coefficients (a = 3,  b = 1), optimized task distribution across processors. This approach minimized processor idle times and reduced inter-tile communication delays. PSOLS, which does not incorporate wave-angle scheduling, exhibited a higher execution time, demonstrating the importance of this feature in PSOALS. Static partitioning methods, such as Block V and Block H, performed poorly because they were unable to adapt to the computationally intensive nature of the tasks. Cyclic scheduling methods (Cyclic V and Cyclic H) demonstrated moderate improvement but were less effective than PSOALS due to their deterministic task allocation strategies. GALS and PSOLS performed well but lacked the adaptability and efficiency introduced by wave-angle scheduling in PSOALS.

#### 5.3.2. Benchmarking in 4 × 6 loop dimensions and 2 processors.

Table 6 presents a performance comparison of the proposed method with existing algorithms for a 4 × 6 task-scheduling problem on two processors. The results further highlight the superior efficiency and adaptability of PSOALS, particularly in environments with balanced computation-to-communication ratios. PSOALS achieved the fastest execution time of 147, outperforming all other methods. Block-based methods (Block V and Block H) recorded execution times of 164 and 179, respectively. Cyclic methods (Cyclic V and Cyclic H) performed slightly better than Block H, achieving times of 164 and 178. GALS and PSOLS, while competitive, were marginally slower than PSOALS, with times of 150 and 147.

**Table 6:** Problem with dimensions 4 × 6 with two processors.

Algorithm inputs		Algorithm execution time										
		Previous methods										
Dimensions of the two-level loop		Method	Block V		Block H	Cyclic V		Cyclic H		GALS	PSOLS	
UB1	4	Execution time	164		179	164		178		150	147	
UB2	6	**Proposed method**										
Parallel system		Execution number		PSOALS			Angle coefficient		Result			
NP	3						a	b				
Vcomp	9	1		147			1	3	Best time		a	b
Vcom1	7	2		147			1	1	147		1	1
Vcom2	7	3		150			1	1	Average		148.2	
Number of iterations		4		150			1	1				
NG	2000	5		147			3	1	Equal			

PSOALS consistently delivered the best results across multiple executions, with minimal variation in execution times. The average execution time for PSOALS was 147, demonstrating its robustness and reliability. Other methods, particularly Block V, exhibited greater variability and higher average times, reflecting their inefficiency in handling task dependencies dynamically. The angle coefficients (a = 1, b = 1) ensured balanced task distribution across processors, reducing idle times and minimizing intertie communication delays. The inclusion of wave angle scheduling enabled PSOALS to achieve optimal task execution sequences, significantly outperforming PSOLS, which does not incorporate this feature.

Block-based methods such as block V and block H struggled to adapt to the dependencies and workload distribution in this experiment, resulting in higher execution times. Cyclic Methods like cyclic V and cyclic H performed better than block-based methods but were less effective than PSOALS due to their inability to dynamically adapt to the task dependencies. GALS and PSOLS methods demonstrated reasonable adaptability, but their lack of wave-angle scheduling mechanisms limited their ability to achieve optimal execution times compared to PSOALS.

#### 5.3.3. Benchmarking in 8 × 4 loop dimensions and 3 processors.

Table 7 evaluates the performance of the proposed method against other algorithms for a 8 × 4 task scheduling problem executed on three processors. The results emphasize the efficiency and adaptability of PSOALS, particularly in handling scenarios with diverse computation and communication requirements. PSOALS achieved the fastest execution time of 176, showcasing superior performance compared to all other methods. Block-based methods (Block V and Block H) resulted in execution times of 229 and 194, respectively, while cyclic methods (Cyclic V and Cyclic H) recorded times of 242 and 194. GALS and PSOLS exhibited competitive performance but were slightly slower than PSOALS, both recording 176.

**Table 7:** Problem with dimensions 8 × 4 with 3 processors.

Algorithm inputs		Algorithm execution time										
		Previous methods										
Dimensions of the two-level loop		Method	Block V		Block H	Cyclic V		Cyclic H		GALS	PSOLS	
UB1	8	Execution time	229		194	242		194		176	176	
UB2	4	**Proposed method**										
Parallel system		Execution number		PSOALS			Angle coefficient		Result			
NP	3						a	b				
Vcomp	9	1		178			1	3	Best time		a	b
Vcom1	7	2		176			1	1	147		1	1
Vcom2	7	3		185			1	1	Average		179	
Number of iterations		4		308			1	1				
NG	2000	5		178			1	1	Equal			

PSOALS demonstrated exceptional consistency in execution times across multiple executions, with an average execution time of 179. This consistency highlights its robustness against stochastic variations and dependency constraints. In contrast, block-based and cyclic methods exhibited higher variability in their results, undermining their reliability for dynamic task scheduling. The angle coefficients (a = 1, b = 1) facilitated balanced task distribution across processors, effectively minimizing idle times and intertile communication delays. The wave-angle scheduling mechanism allowed PSOALS to achieve optimized execution sequences, significantly outperforming methods like PSOLS, which do not utilize this feature. Block-based methods such as block V and block H suffered from poor adaptability to task dependencies, resulting in higher execution times. While cyclic methods such as cyclic V and cyclic H improved upon the performance of block-based methods, they lacked the dynamic adaptability necessary for optimal scheduling, yielding suboptimal execution times. GALS and PSOLS despite their relatively strong performance, these methods were unable to match the efficiency of PSOALS, primarily due to the absence of wave-angle scheduling.

#### 5.3.4. Benchmarking in 10 × 6 loop dimensions and 3 processors.

Table 8 evaluates the performance of the proposed method against other algorithms for a 10 × 6 task scheduling problem executed on three processors. The results underscore the effectiveness of PSOALS in managing complex computation and communication scenarios. PSOALS achieved the fastest execution time of 308, significantly outperforming other methods. Block-based methods (Block V and Block H) recorded execution times of 413 and 329, respectively. Cyclic methods (Cyclic V and Cyclic H) demonstrated better results than Block V, with execution times of 326 and 370, respectively. GALS and PSOLS were competitive, both achieving a execution time of 308. In terms of stability across iterations, PSOALS demonstrated minimal variability across multiple executions, maintaining an average execution time of 314. This consistent performance highlights the robustness and reliability of PSOALS in handling varying computational loads. Other methods, such as Block V and Cyclic H, showed greater variability and less stability in execution times.

**Table 8:** Problem with dimensions 10 × 6 with 3 processors.

Algorithm inputs		Algorithm execution time										
		Previous methods										
Dimensions of the two-level loop		Method	Block V		Block H	Cyclic V		Cyclic H		GALS	PSOLS	
UB1	10	Execution time	413		329	326		370		308	308	
UB2	6	**Proposed method**										
Parallel system		Execution number		PSOALS			Angle coefficient		Result			
NP	3						a	b				
Vcomp	9	1		318			1	3	Best time		a	b
Vcom1	7	2		320			1	1	308		1	1
Vcom2	5	3		312			1	1	Average		314	
Number of iterations		4		308			1	1				
NG	2000	5		312			1	1	Equal			

The angle coefficients (a = 1, b = 1) optimized task distribution across processors, thereby reducing idle time and minimizing inter-tile communication delays. Wave angle scheduling enabled PSOALS to achieve superior task execution efficiency compared to methods like PSOLS, which lack this feature. Block-based methods (block V and block H) struggled to adapt to dynamic dependencies, resulting in higher execution times and poor adaptability. Cyclic V and Cyclic H performed better than block-based methods but lacked the ability to dynamically adapt to task dependencies, leading to suboptimal results. GALS and PSOLS While these methods showed strong performance, their lack of wave-angle scheduling limited their ability to achieve the optimal execution times observed with PSOALS. In order to evaluate the performance of the suggested method, other experiments have been performed; the results are outlined by Tables 9, 10, 11. Consistent with prior experiments, the results confirm the superiority of the proposed method over the previous method.

**Table 10:** Problem with dimensions 10 × 10 with three processors.

Algorithm inputs		Algorithm execution time										
		Previous methods										
Dimensions of the two-level loop		Method	Block V		Block H	Cyclic V		Cyclic H		GALS	PSOLS	
UB1	10	Execution time	240		228	440		440		308	224	
UB2	10	**Proposed method**										
Parallel system		Execution number		PSOALS			Angle coefficient		Result			
NP	3						a	b				
Vcomp	2	1		200			5	6	Best time		a	b
Vcom1	10	2		200			5	6	64		5	6
Vcom2	10	3		200			5	6	Average		200	
Number of iterations		4		200			5	6				
NG	2000	5		200			5	6	improve			

**Table 11:** Problem with dimensions 9 × 4 with 3 processors.

Algorithm inputs		Algorithm execution time										
		Previous methods										
Dimensions of the two-level loop		Method	Block V		Block H	Cyclic V		Cyclic H		GALS	PSOLS	
UB1	9	Execution time	286		191	196		191		187	186	
UB2	4	**Proposed method**										
Parallel system		Execution number		PSOALS			Angle coefficient		Result			
NP	3						a	b				
Vcomp	10	1		186			1	1	Best time		a	b
Vcom1	1	2		186			1	1	186		1	2
Vcom2	1	3		186			1	1	Average		186.4	
Number of iterations		4		187			1	1				
NG	2000	5		187			1	1	equal			

**Table 9:** Problem with dimensions 9 × 7 with 4 processors.

Algorithm inputs		Algorithm execution time										
		Previous methods										
Dimensions of the two-level loop		Method	Block V		Block H	Cyclic V		Cyclic H		GALS	PSOLS	
UB1	9	Execution time	282		240	240		240		237	237	
UB2	7	**Proposed method**										
Parallel system		Execution number		PSOALS			Angle coefficient		Result			
NP	4						a	b				
Vcomp	10	1		237			1	1	Best time		a	b
Vcom1	1	2		237			1	1	237		1	1
Vcom2	1	3		238			1	1	Average		237.6	
Number of iterations		4		238			1	1				
NG	2000	5		238			1	1	Equal			

Generally, the PSO can be used to sort out different continuous and discrete optimization algorithms. However, PSO does not perform well in some problems. PSO is inefficient in problems with a large number of variables, with sharp local optima, and with strict constraints. In this study, a specific variant of the hybrid PSO was developed and adapted for loop scheduling. This study employs a hybrid PSOALS, a novel approach designed to dynamically parallelize nested loops in heterogeneous IoT environments. PSOALS makes an effective loop scheduling (workloads balancing, adapting the resource constraints, and enhancing parallelism) in the programs related to IoT applications. The experimental results of demonstrate the efficiency of the suggested method in the loop parallelization problem. The scalability and applicability of the suggested method for the energy-aware and real-time IoT platforms can be taken into consideration as future studies.

**Fig 15:**
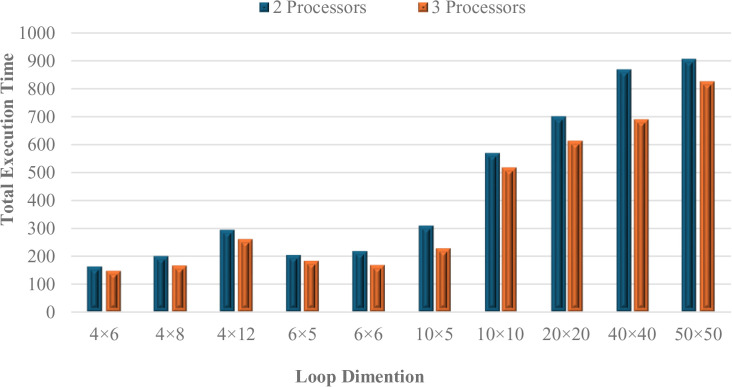
Evaluation of the suggested loop parallelization method in different loop dimensions in terms of execution time.

As depicted in Fig 15, comparing execution times for 2 processors and 3 processors, it is evident that increasing the number of processors reduces the execution time significantly. This highlights the efficiency of the parallelization method (PSOALS), particularly in scenarios with higher computational loads. The execution time difference between the two configurations becomes more pronounced as loop dimensions expand, demonstrating the scalability of the method. With respect to the scalability criterion, for smaller loop dimensions (4 × 6 and 4 × 8), the execution time difference between 2 and 3 processors is less noticeable. However, as loop dimensions reach 40 × 40 and beyond, the advantage of using additional processors becomes stark. This implies that the suggested PSOALS is particularly advantageous and scalable for tasks involving larger loop dimensions, where computational efficiency is critical. The findings suggest that the suggested loop parallelization method is highly effective and scalable, especially when applied to larger and more complex loop structures. To maximize efficiency, implementing this method with a higher number of processors is recommended for applications with demanding computational requirements.

One of the core strengths of PSOALS is its wave-angle scheduling mechanism, which accounts for data dependencies and communication delays in a directional manner. This allows the algorithm to balance load and reduce synchronization bottlenecks; balance load and synchronization bottlenecks are two essential factors in distributed IoT nodes that rely on wireless communication or low-bandwidth links. Furthermore, by encoding loop iterations as particles and incorporating dependency-aware fitness functions, PSOALS ensures that execution order adheres to correctness constraints while still optimizing performance metrics.

Adapting PSOALS to IoT platforms involves configuring the algorithm to consider platform-specific constraints such as core availability, processor heterogeneity, and memory limitations. This can be achieved through parameter tuning within the PSOALS framework (adjusting population size, communication velocity, and fitness weights) to reflect the characteristics of the underlying hardware. For instance, in resource-constrained nodes, the algorithm can prioritize efficient scheduling by penalizing high-communication paths or balancing workload across low-power cores. Similarly, real-time IoT applications can benefit from adjusting the convergence speed and iteration limits to meet deadline constraints. The hybrid nature of PSOALS, combining PSO, GA, and diversity-inducing techniques, makes it robust to varying workloads and adaptable to the non-deterministic behavior of real-world IoT systems.

## 6. Threats to validity

While the developed PSOALS method significantly advances IoT task scheduling, several critical challenges remain unresolved. This section identifies six primary open issues that warrant further investigation to extend the capabilities of the proposed framework.

### 6.1. Scalability to large-scale IoT deployments

While working in parallel and during training, you need to start analyzing and optimizing before completing the test. 3B or higher, the higher the number of people, the more likely the task is to be completed quickly. By connecting with the people you need, you can use parallel tools to complete the work. Therefore, it is important that you no longer have to worry and that the device is no longer needed. PSOALS was developed to enable the current IoT experience. In addition, IoT applications and millions of people use connected objects, connected objects, objects, and selected tasks that help me build a new life. Mevcut, however, was sure that this was not the case because I didn’t know this would be the case. The products used must be up-to-date and therefore, appear as if the products were mixed. It is important to improve the algorithm and optimize the IoT system.

### 6.2. Adaptability to dynamic resource availability

The proposed PSOALS method assumes static resource availability, which limits its adaptability in real-world applications. Enhancing the framework to account for dynamic changes in network topology and resource availability is critical. Techniques such as predictive modeling of resource states or incorporating real-time feedback mechanisms can ensure that the scheduling system remains resilient and efficient under fluctuating conditions.

### 6.3. Energy-aware scheduling for IoT devices

Energy efficiency is a key concern in IoT environments, where many devices operate on limited battery power. The PSOALS framework prioritizes execution time minimization but does not explicitly address energy consumption. Future enhancements should integrate energy-aware scheduling strategies that optimize both computational and communication energy usage. Balancing energy consumption with performance objectives can extend device lifetimes and reduce the overall energy footprint of IoT systems, particularly in scenarios where energy constraints are critical, such as remote monitoring or wearable devices.

### 6.4. Real-time task scheduling with deadlines

Many IoT applications, such as industrial automation, healthcare monitoring, and autonomous vehicles, require strict adherence to task deadlines. The current PSOALS framework focuses on minimizing makespan but lacks explicit mechanisms for handling tasks with real-time deadlines. Introducing deadline-aware scheduling capabilities would enable the framework to prioritize urgent tasks and ensure timely execution, even under high system loads. This enhancement is crucial for extending the applicability of PSOALS to time-sensitive IoT domains.

### 6.5. Integration with heterogeneous IoT devices

IoT networks are highly heterogeneous, comprising devices with diverse computational capabilities, energy constraints, and communication protocols. The current PSOALS implementation assumes homogeneous resources, which may not align with real-world deployments. Extending the framework to accommodate device heterogeneity requires adaptive scheduling algorithms that can dynamically allocate tasks based on the specific capabilities and constraints of each device. This enhancement would make the method more robust and versatile in practical IoT environments.

### 6.6. Experimental validation in real-world scenarios

While the PSOALS framework has been tested in simulation environments, real-world validation is crucial to assess its practicality and effectiveness under realistic conditions. Real-world IoT deployments introduce unpredictable factors such as network latencies, hardware failures, and environmental variations. Implementing and evaluating PSOALS in diverse IoT scenarios, such as smart homes, industrial IoT, or urban infrastructure, will provide valuable insights and identify potential limitations that simulations may not reveal. This step is essential for ensuring the framework’s readiness for practical adoption.

## 7. Conclusion and future work

Efficient task scheduling in IoT environments is vital for optimizing execution time, resource utilization, and scalability. This paper proposed PSOALS, an innovative scheduling framework that combines PSO with wave-angle scheduling. The method effectively resolves complex nested loop dependencies, minimizes communication overhead, and balances workload distribution across heterogeneous IoT devices. Experimental evaluations demonstrated the superiority of PSOALS over existing methods, including block-based, cyclic, and hybrid PSO approaches, in terms of convergence speed, stability, and reliability. PSOALS uses dynamic task prioritization and dependency-aware tiling strategies to improve execution efficiency, making it an excellent fit for small- to medium-scale IoT systems. While the proposed method addresses key challenges in IoT scheduling, several open issues remain for future exploration. Scalability to large-scale IoT deployments is a critical next step, requiring decentralized or hierarchical architectures to manage the increasing complexity of tasks and dependencies. Incorporating energy-aware scheduling will ensure efficiency in power-constrained devices, extending their operational lifetimes.

Future work can focus on integrating real-time scheduling capabilities to handle tasks with strict deadlines, enhancing the framework’s applicability in time-sensitive domains like healthcare and industrial automation. Additionally, the heterogeneity of IoT devices and the dynamic availability of resources in real-world deployments necessitate adaptive algorithms that respond to environmental changes. Using other hybrid metaheuristic algorithms [18–20] for loop parallelization may yield better results. Finally, real-world validation in diverse IoT scenarios will be conducted to assess the framework’s robustness and practical utility, paving the way for its adoption in large-scale, dynamic IoT ecosystems.
